# Léiomyosarcome de la veine cave inférieure: un cas clinique

**DOI:** 10.11604/pamj.2015.20.283.6449

**Published:** 2015-03-24

**Authors:** Nabil Hammoune, Faycal El Guendouz, Siham Elhaddad, Rachida Latib, Imane Chami, Najib Boujida, Abdelaziz Hommadi

**Affiliations:** 1 Service de Radiologie, Troisième Hôpital Militaire, Laayoune, Maroc; 2 Service d'Endocrinologie, Troisième Hôpital Militaire, Laayoune, Maroc; 3 Service de Radiologie, Centre Hospitalier Universitaire Ibn Sina, Rabat, Maroc; 4 Service de Radiologie, Institut National d'Oncologie, Rabat, Maroc

**Keywords:** Léiomyosarcome, veine cave inférieure, imagerie, Leiomyosarcoma, inferior vena cava, imagery

## Abstract

Le léiomyosarcome de la veine cave inférieure est une tumeur maligne rare développée aux dépens des cellules musculaires lisses de la paroi vasculaire. L'imagerie radiologique par tomodensitométrie ou résonance magnétique nucléaire est un élément important au diagnostic et au bilan d'extension tumorale. Le traitement est chirurgical. Nous illustrons l'apport de l'imagerie à travers un cas de léiomyosarcome révélé par des épigastralgies paroxystiques, dont le diagnostique était orienté par la TDM, et confirmé par l’étude histologique. L’évolution était favorable. Après 2 ans de recul, la patiente était indemne de toute récidive tumorale.

## Introduction

Le léiomyosarcome (LMS) de la veine cave inferieure (VCI) est une tumeur maligne rare développée aux dépens des cellules musculaires lisses de la paroi vasculaire. Il s'agit d'une pathologie longtemps asymptomatique ce qui explique les retards diagnostiques importants au stade tumoral extravasculaire. L'imagerie en coupe est un élément important au diagnostic et au bilan de l'extension tumorale. Le traitement en est chirurgical par l'exérèse large afin de réduire le risque de récidive locorégionale. Nous illustrons l'apport de l'imagerie à travers un cas de léiomyosarcome révélé par des épigastralgies paroxystiques, dont le diagnostique était orienté par la TDM, et confirmé par l’étude histologique. L’évolution était favorable. Après 2 ans de recul, la patiente était indemne de toute récidive tumorale.

## Patient et observation

**Figure 1:**
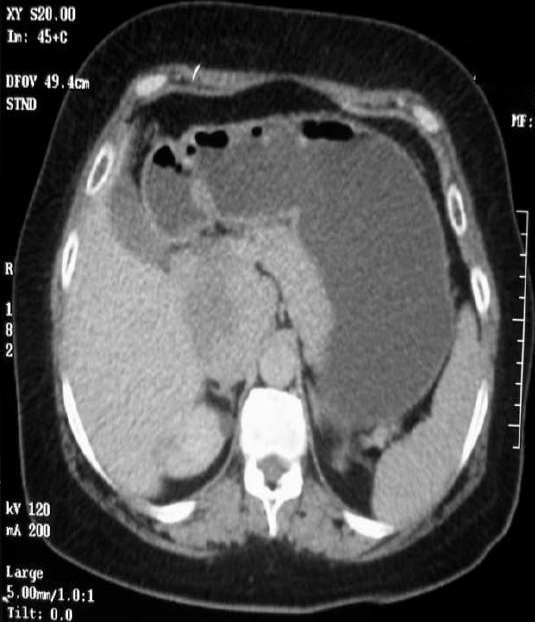
TDM abdominopelvienne en coupes axiales avant injection de produit de contraste montrant une masse tissulaire rétro-péritonéale latéralisée à droite, lobulée, hypodense et mesurant 62x60x120mm

**Figure 2:**
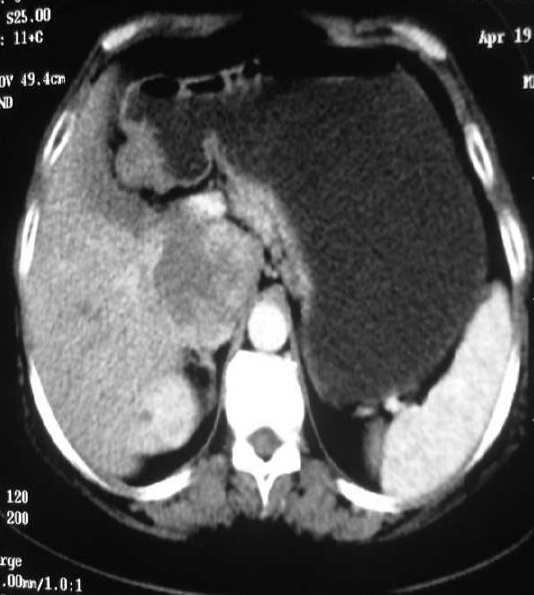
TDM abdominopelvienne en coupes axiales après injection de produit de contraste montrant un rehaussement hétérogène de la masse tissulaire rétro-péritonéale au dépend de la veine cave inferieure (VCI) rétrohépatique, elle englobe la surrénale et arrive au contact de la tête du pancréas

Femme de 55 ans, ayant comme antécédents une appendicectomie, qui a présenté depuis 8 ans des douleurs épigastriques paroxystiques isolées non améliorées par les traitements symptomatiques. L'examen clinique a trouvé une masse dure de l’épigastre non battante, non sensible, mobile par rapport au plan superficiel sans hépatomégalie, ni splénomégalie ni adénopathie associée. L’échographie abdominale a révélé un processus hypoéchogène hétérogène, polycyclique de siège épigastrique. Pour une meilleure caractérisation de la masse, un complément scannographique thoraco-abdomino-pelvien, sans et avec injection de produit de contraste a été réalisé. Il a précisé le siège rétro-péritonéale latéralisée à droite de la masse, au dépend de la veine cave inferieure (VCI) rétrohépatique, qui est de nature tissulaire lobulée hypodense, rehaussée de façon hétérogène, mesurant 62x60x120mm. Elle englobe la surrénale droite et arrive au contact de la tête du pancréas, sans signes d'envahissement évident ou de localisation secondaire associée (Figure 1 et Figure 2). La patiente a bénéficié d'une exérèse de la masse en bloc. L’étude anatomopathologique était en faveur d'un léiomyosarcome de la VCI et les suites étaient favorables après 2 ans de surveillance.

## Discussion

Les léiomyosarcomes à point de départ vasculaire sont rares, la VCI représente leur siège d’élection (38%) [1], cette localisation est décrite pour la première fois par Perl en 1871 [2], les données publiées de Léiomyosarcomes de la VCI dans une base de données mondiale sont limitées [3]. Le LMS de la VCI se définit comme une tumeur maligne mésenchymateuse développée au dépend des fibres musculaires lisses de la paroi veineuse [4]. La division de la VCI en 3 segments permet de classer les lésions en segment I strictement sous-rénal; segment II comprenant l'origine des veines rénales et le segment rétrohépatique de la VCI; enfin segment III comprenant l'origine des veines sus-hépatiques et le segment supra-hépatique de la VCI jusqu’à l'oreillette droite [5]. La localisation la plus fréquente concerne le segment II comme c'est le cas de notre observation. Il touche l'adulte avec une nette prédominance féminine (82%), son incidence serait plus élevée chez les sujets immunodéprimés, où il est associé au virus Epstein-Barr [2, 6]. Ces tumeurs se caractérisent par leur croissance lente, leur caractère peu infiltratif et leur potentiel métastatique tardif qui se fait plutôt par voie systémique [5], ce qui va se traduire par une symptomatologie clinique longtemps peu significative, expliquant le retard du diagnostic. Cette dernière dépend surtout de leur localisation et de l´importance de leur développement local. Cette tumeur est révélée souvent par des douleurs abdominales, une masse abdominale peu sensible, une perte de poids, des nausées, une fébricule et/ou des œdèmes des membres inférieurs [6]. L'absence d’œdème des membres inférieurs signe habituellement le développement d'une circulation veineuse collatérale de suppléance [7]. L’échographie montre habituellement une tumeur rétropéritonéale à contours lobulés, hétérogène. Elle précise sa situation dans un segment anatomique de la VCI et son extension aux organes de voisinage. Le doppler renseigne sur la perméabilité de la VCI, et du réseau veineux de voisinage (des veines de voisinage, veines rénales, veine porte, veines sus hépatiques) [6].

La TDM après injection de produit de contraste permet de préciser la nature de la masse, souvent de contours polylobés, présentant un rehaussement hétérogène après injection de produit de contraste. Il détermine la localisation, les rapports avec les organes de voisinage, le degré d'obstruction de la VCI et des veines de voisinage. Elle permet également la recherche de métastases notamment hépatiques et pulmonaires [2]. L'IRM n'apporte pas d’éléments nouveaux par rapport aux nouvelles générations de scanner [6, 7], elle montre la tumeur en signal intermédiaire sur les séquences T1 qui se renforce en T2, elle permet de préciser la topographie de la tumeur, ses rapports avec les organes de voisinage, et son retentissement sur la lumière de la VCI en différenciant les envahissements néoplasiques des thromboses cruoriques. Les techniques invasives (angiographie, échographie endo-vasculaire) ont perdu de leur intérêt dans le diagnostic des LMS de la VCI du fait du développement des techniques non invasives [7]. La confirmation du diagnostic est apportée par l'analyse histologique ou cytologique des fragments de biopsie. Ils peuvent être obtenus soit par biopsie échoguidée ou scanoguidée, soit parfois par biopsie transjugulaire quand la tumeur fait saillie dans la lumière de la veine. L'histologie révèle une prolifération fasciculée de cellules allongées, fusiformes, en faisceaux tourbillonnants dont le caractère malin est retenu sur l'aspect des noyaux [1]. Les diagnostics différentiels d'une masse rétropéritonéale affectant la VCI incluent le Léiomyosarcome, le sarcome intimal, le thrombus tumoral de drainage veineux, les tumeurs stromales gastro-intestinales et le thrombus cruorique, les tumeurs surrénaliennes (phéochromocytome) rénales étendues à la VCI et les paragangliomes [3]. D'un point de vue thérapeutique, le seul traitement dont l'impact sur la survie ait été démontré reste la chirurgie radicale, dominé par l´exérèse monobloc de la tumeur, élargie éventuellement aux organes de voisinage, ce traitement est à adapter à la topographie de la tumeur, le but du traitement est l'exérèse complète de la tumeur, la conservation du retour veineux et la prévention des récidives [3, 4, 7]. la place des traitements adjuvants de type radiothérapie et chimiothérapie reste à définir [5, 7]. Le pronostic des LMS de la VCI reste sombre avec un taux de survie à 5 ans inférieur à 50% et un taux de survie à 10ans inférieur à 30%. L’évolution est inquiétée par la survenue des métastases et par la récidive qui survient dans 30% à 50% des cas, avec une médiane de survie à 25 mois [5]. Vu l'accessibilité de la tumeur à la chirurgie, notre patiente a bénéficié d'une exérèse complète, l’évolution a été marqué par l'absence de récidive et de métastase au terme d'une surveillance de deux ans.

## Conclusion

Les léiomyosarcomes de la VCI sont rares, de diagnostic souvent tardif et donc de pronostic sombre. Les moyens radiologiques actuels devraient permettre leur diagnostic plus précoce. Le seul traitement efficace est l'exérèse chirurgicale, le pronostic est fonction du stade au moment du diagnostic.
